# Pattern of substance use and substance use disorder in adolescent learners at public secondary schools in Gaborone, Botswana

**DOI:** 10.1371/journal.pone.0268961

**Published:** 2022-09-09

**Authors:** Anthony A. Olashore, Saeeda Paruk, Taboka Maphorisa, Boitshepo Mosupiemang

**Affiliations:** 1 Faculty of Medicine, Department of Psychiatry, University of Botswana, Gaborone, Botswana; 2 Department of Psychiatry, Nelson R Mandela School of Medicine, University of KwaZulu-Natal, Durban, South Africa; 3 Department of Psychology, University of Botswana, Gaborone, Botswana; Rutland Regional Medical Center, UNITED STATES

## Abstract

**Introduction:**

Substance use amongst adolescents remains a global public health challenge. The potential negative health outcomes of substance use suggest the need to understand the pattern of use and the associated factors among adolescents. This study aimed to describe the prevalence of substance use, SUDs, and PD and the associated factors in adolescent learners at public schools in Gaborone, Botswana.

**Methods:**

A cross-sectional study was conducted at 13 public secondary schools in Gaborone among 742 students. Assessment tools included the World Health Organization drug questionnaire, DSM-5 interview for SUD, and the General Health Questionnaire-12.

**Results:**

The mean age (SD) of the participants was 15.26 (1.57) years, and there were more females (55.5%). Over two-fifths (44.6%) of learners reported psychoactive substance use in the past 12 months, and 31.5% meeting DSM 5 criteria for a SUD. Alcohol was the most used psychoactive substance (25.1%). Male gender (AOR = 1.94; 95% CI: 1.26–2.995), having a friend (AOR = 4.27; 95% CI: 2.68–6.78), or father (AOR = 1.87; 95% CI: 1.14–3.04), who uses substance, and higher levels of PD (AOR = 1.09; 95% CI: 1.03–1.17) remained significantly associated with SUD. Regular participation in religious activities negatively correlated with SUD (AOR = 0.61; 95% CI: 0.38–0.96).

**Conclusion:**

The high prevalence of substance use and SUDs among in-school adolescents is concerning. Substance use programs need to include family-focused and religious-based therapy and youth empowerment in developing positive peer relationships. Also, they should be integrated with mental health screening to assess comorbid PD.

## Introduction

According to the United Nations Department of Economic and Social Affairs Disability, mental health conditions, including alcohol and drug-related problems, are the tenth leading cause of dysfunction in developed and developing countries [1]. Alcohol is the most used substance in most settings, followed by tobacco and cannabis [2–4]. Data from the 2018 National Survey on Drug Use and Health in the United States of America (USA) indicated that, in 2017, over 20.3 million people aged 12 and older presented with substance use disorders (SUDs), and of whom 14.8 million were attributed to alcohol use [5]. In another study conducted in the USA, 26% of youths met the DSM-5 criteria for an alcohol use disorder, while 15% admitted to having problems with cannabis use [6].

Substance use disorder (SUD) is defined as the persistent use of drugs despite significant harmful consequences [7]. It is associated with negative health outcomes and problems at work, school, or at home [8]. It is a new term used by the Diagnostic and Statistical Manual of Mental Disorders, fifth edition (DSM-5) to represent drug abuse and dependence [7].

Little is known about factors that may lead to the development of SUDs in adolescents [6]. Studies have investigated the possible risk factors associated with substance use and SUDs in adult populations [9]. In a study conducted in Suriname’s (South America) urban and rural areas, men were more likely to develop SUD than women [9]. Being married and having a higher education were protective, while the lower-income occupation was associated with increased SUD [9]. Another study from the US reported the association between low economic status and lack of employment in adults with SUD [10].

Studies previously conducted in Botswana among secondary school students also revealed that boys were more likely to have alcohol and drug problems, albeit the DSM-5 criteria for SUD were not used [11,12]. On the contrary, no gender difference was found in another study conducted among first-year university students in Botswana [13]. However, parental involvement in psychoactive substances and association with drug-using peers correlated with cannabis use disorder, while frequent religious participation was protective [14].

Botswana is one of the upper-middle-income countries in sub-Saharan Africa, with a relatively good healthcare system and a predominantly youthful population [15]. However, there are limited facilities to cater to adolescents with alcohol and drug problems [16]. This could be related to the limited data on the extent of alcohol and drug use and its outcomes on physical and mental health in this age group. Therefore, this present study intended to fill this gap in knowledge by determining the prevalence of substance use, SUDs, and psychological distress and describing the related factors in adolescent learners in public schools in Gaborone, Botswana.

## Materials and methods

This cross-sectional study involved learners aged 12 to 19 years from 13 selected public secondary schools in Gaborone. The location was chosen because, as Botswana’s capital city, it accounts for more than 10% of the population, is diverse, and has a good blend of Botswana ethnic groups, including foreign nationals. The sample size required for assessing the psychoactive SUDs in the students was determined using Cochran’s formula (N = Z^2^pq/d^2^) for minimum sample size determination in a cross-sectional study. ‘N’ is the minimum sample size required for the study. P = 58.4%, is the proportion of college students who admitted to using substance in a previous study in Botswana [17]. ‘q’ = 1-p = 1–0.584 = 0.416; d = Error margin (Precision) desired, set at 5% = 0.05. Z = Standard normal deviate, set at 95 percent confidence level. (Z = 1.96). Applying the formula: n = 0.93329367/0.0025 = 373.3. The non-response of 30%, 373.3 = 111.99. Therefore, the minimum sample size used for this study was (373 +111.99) 485.29, approximating 500 participants, but 750 students were interviewed.

The participants were from four senior and nine junior secondary schools in Gaborone, Botswana. Of the 750 participants, 300 were from the senior schools, while the remaining 450 were from the junior schools. This proportional allocation was based on the number of grade levels, with three (forms 1–3) in junior school and two (forms 4 and 5) in senior school.

### Sampling technique

A multi-stage sampling design was used for the present study. Nine of the thirteen junior schools were randomly selected as primary sampling sites, while all the four senior secondary schools were included to produce a representative sample of all the government junior and senior secondary schools in Gaborone. The second stage was a random selection of classrooms within the selected secondary schools. In Botswana, the junior secondary schools have three grades (forms 1–3), while the senior schools have two grades (forms 4 and 5). Each grade may have up to three or more classes, labeled ’a’ ’b’ ’c’ or more. The classes in each school were ordered by grade, and at least one class was randomly selected from each grade. However, more than one class was selected from grades with more classes. Whilst we selected 50 students from each of the nine junior schools, 75 participants were randomly selected from each of the four senior schools to represent older students. The third stage involved a random selection of ten to fifteen students (depending on the size of the classes) from the class registers in the junior schools and 20–25 students from the senior schools. For all the stages of selection, the Statistical Package for Social Sciences (SPSS for Windows) was used. For example, the school list or the students’ numbers were entered into the statistical software and was used to generate the desired number of participants.

### Study procedure

All eligible and consenting students were approached during their lunch break or at a time specified as convenient for them. They were assured of confidentiality and anonymity. Data were collected from December 2019 to October 2020. The specified guidelines were strictly followed for those whose interviews were conducted during the COVID-19 pandemic.

The tools used included the modified version of the 37-item World Health Organization (WHO) drug questionnaire, the DSM-5 interview for SUD, and the General Health Questionnaire-12 (GHQ-12). The tools were self-administered to the participants during break time while seated in exam-like conditions but without their class teachers. The instructions were provided, and the research assistants were available to assist those who had difficulty completing the questionnaires. The research assistants administered the DSM-5 interview for SUD to only those who screened positive for using any psychoactive substance in the past 12 months. Finally, those who required help for SUD or psychological distress were referred through their class teachers or parents to the nearest clinic without breaching confidentiality.

### Measures

The first set of instruments, namely the WHO drug questionnaire and GHQ-12, were thus self-administered and completed in less than 30 minutes, while the DSM-5 SUD interview took an additional 10–15 minutes.

**The World Health Organization drug questionnaire:** [18]: The WHO created this tool with the United Nation’s Division of Narcotics, the International Narcotics Control Board, and the International Council on Alcohol and Addictions. It was created to aid in collecting epidemiological data on drug abuse in many parts of the world. It is suggested for use on students and other groups and includes a wide range of psychoactive/illicit substances such as cocaine, cannabis, hallucinogens, opioids, and sedatives. For simplicity, specific examples pertinent to this setting were given for the drug classes. Wherever possible, the common names for these compounds were utilized. For example, in amphetamine-type stimulants (ATS), drugs like methylphenidate (Ritalin), khat, and crystal-meth were mentioned. Participants had the option to add other types of substances that were not included if required. The list of psychoactive chemicals was expanded to include a fictitious drug called ’Relvine.’ to reduce the over-reporting bias, and those participants admitting to using ’relvine’ were omitted from further analysis.

This tool assesses the psychoactive drug usage in one’s lifetime, 12 months, and currently (within the previous 30 days). The current study only looked at the use in the past 12 months. Considering possible interference by the COVID-19 pandemic on substance use patterns, the current use was excluded from the analysis.

The WHO drug questionnaire also includes questions about the respondents’ socio-demographic characteristics such as age, ethnicity, religious participation, parents’ marital status, level of education, and occupation. For example, religious participation was measured by the frequency with which people attended religious activities. Subjective responses such as ’never,’ ’rarely,’ sometimes,’ ’often,’ and ’regularly’ were used. These responses were recategorized as ’never’ or ’rarely’ and ’sometimes’ to ’regularly’ for easy analysis. The parents’ occupation or employment status was classified based on the 2008 Botswana Standard Classification of Occupations [19]. It was organized into three groups: i) The upper occupational group includes legislators, managers, professionals, and large-scale traders. ii) The lower group includes the plant operators, clerks, cleaners, small-scale traders, and technicians. iii) The unemployed.

**The DSM-5** [7] diagnostic criteria were utilized to further assess SUD among those who had taken any psychoactive substance in the last 12 months. It combines the DSM-IV categories of substance abuse and dependence into a single disorder. SUD has a wide variety of problems resulting from substance use and comprises 11 different criteria, of which two are required for a diagnosis. In addition, it has specifiers for severity and whether one is in early or late remission.

**The General Health Questionnaire-12 (GHQ-12)** [20]: was used to evaluate whether the students expressed any psychological distress. It consists of 12 items, each assessing the severity of a psychological problem in the past few weeks using a 4-point Likert-type scale (from 0 to 3). In the present study, the respondents were expected to single out their best fit in the past few weeks. A ’0’ score was given if the respondent selected the first two responses and a score of ’1’ for the last two. For each question, a score of ’0’ was considered negative and ’1’ positive. A positive score is suggestive of the presence of psychological distress. This tool generates a total score ranging from 0 to 36. As previously reported in Botswana and Nigeria, a score of 3 and above indicates psychological distress [13,21]. This tool has been used in different continents and has been found to have good internal consistency, as shown by Cronbach’s alpha range of 0.82–0.89.

### Data analysis

Data Analysis was conducted using the Statistical Package for Social Sciences (SPSS, Version 21). Descriptive statistics: the mean was used to describe the continuous socio-demographic variables such as age, GHQ score, and monthly allowance, while percentages were used for the categorical variables like gender, religion, and ethnicity. The alcohol and drug use patterns were represented with a bar chart and pie chart. SUD, the outcome variable, was defined according to DSM-5: participants who had used any substance in the past 12 months and met the DSM-5 criteria for the substance were said to have SUD. Chi-square tests were used to show the relationships between SUD and all the categorical variables, e.g., gender, while the independent t-tests were performed to explore the relationship between the continuous variables such as age and SUD. All the variables with a p-value less than 0.25 were entered into a logistic regression model to explore these relationships further, with SUD as the dependent variable. A multicollinearity test was performed to check for high intercorrelations among the predictors, and those with a tolerance value of less than 0.1 were regarded as highly correlated. The Hosmer-Lemeshow goodness of fit test for logistic regression was also conducted, and the level of statistical significance for all tests was set at p< 0.05.

### Ethical considerations

Before the data collection, approval was sought from the University of Botswana Research and Ethics Review Committee, the Ministry of Education, and the relevant school authorities. In addition, written informed consent was sought from the parents of all the selected students. Only those students who returned their signed consent forms and approved in writing were interviewed.

## Results

### Socio-demographic characteristics of the respondents

Seven hundred and fifty secondary school students participated in the survey during the study period; seven hundred and forty-two (98.9%) of the questionnaires retrieved were analyzed. Eight questionnaires were discarded due to incomplete responses in the variable of interest and because some admitted to using the fictitious drug, which was intentionally added to reduce overreporting. Table 1 depicts the socio-demographic and general health questionnaire scores of the participants. The participants’ mean age (SD) was 15.26 (1.57) years. There were more female students (55.5%) participants in the study than males. Most of the students (80.6%) were from the Tswana ethnic group and were Christians (89.5%).

**Table 1:** Demographic characteristics of the participants.

Variable	Statistic	
**Age, years; mean (SD)**	**15.26(1.57)**	
**General health questionnaire score (SD)**	**3.28(3.26)**	
**Monthly allowance in USD (SD)**	**19.60(19.380)**	
	**Frequency N**	**%**
**Gender**	**733** *	**100**
Male	326	44.5
Female	407	55.5
**Religion**	**735** *	**100**
No religious affiliation	58	7.9
Christianity	658	89.5
Islam	4	0.5
Others**	15	2.0
**Frequency of religious participation**	**683** *	**100.0**
Rarely or never	169	24.7
Often to regularly	514	75.3
**Ethnic group**	**713** *	**100**
Non-citizen	1	0.1
Tswana	575	80.6
Kalanga	100	14.0
Other ethnic groups^#^	37	5.2
**Have you lost a parent to death or marital separation?**	**736** *	**100**
No	18	2.4
Yes	718	96.8
**Fathers’ occupation/employment status**	**596** *	**100**
Unemployed	125	21.0
Higher occupational level	237	39.8
Lower occupational level	234	39.3
**Mothers’ occupation/employment status**	**628** *	**100**
Unemployed	180	28.7
Higher occupational level	268	42.7
Lower occupational level	180	28.6
**Does your father take any substance?**	**655** *	**100**
No	413	63.1
Yes	242	36.9
**Does your mother take any substance?**	**708** *	**100**
No	549	77.5
Yes	159	22.5
**Do you have a friend taking any substance?**	**716** *	**100**
No	548	76.5
Yes	168	23.5

*N = n not equal to 742 due to missing data, not applicable, or don’t know.

**Others = African traditional religion, and Hinduism and Buddhism.

^#^ Other ethnic groups such as Basarwa and Kgalagadi.

### Prevalence and pattern of psychoactive substance use

**Fig 1:**
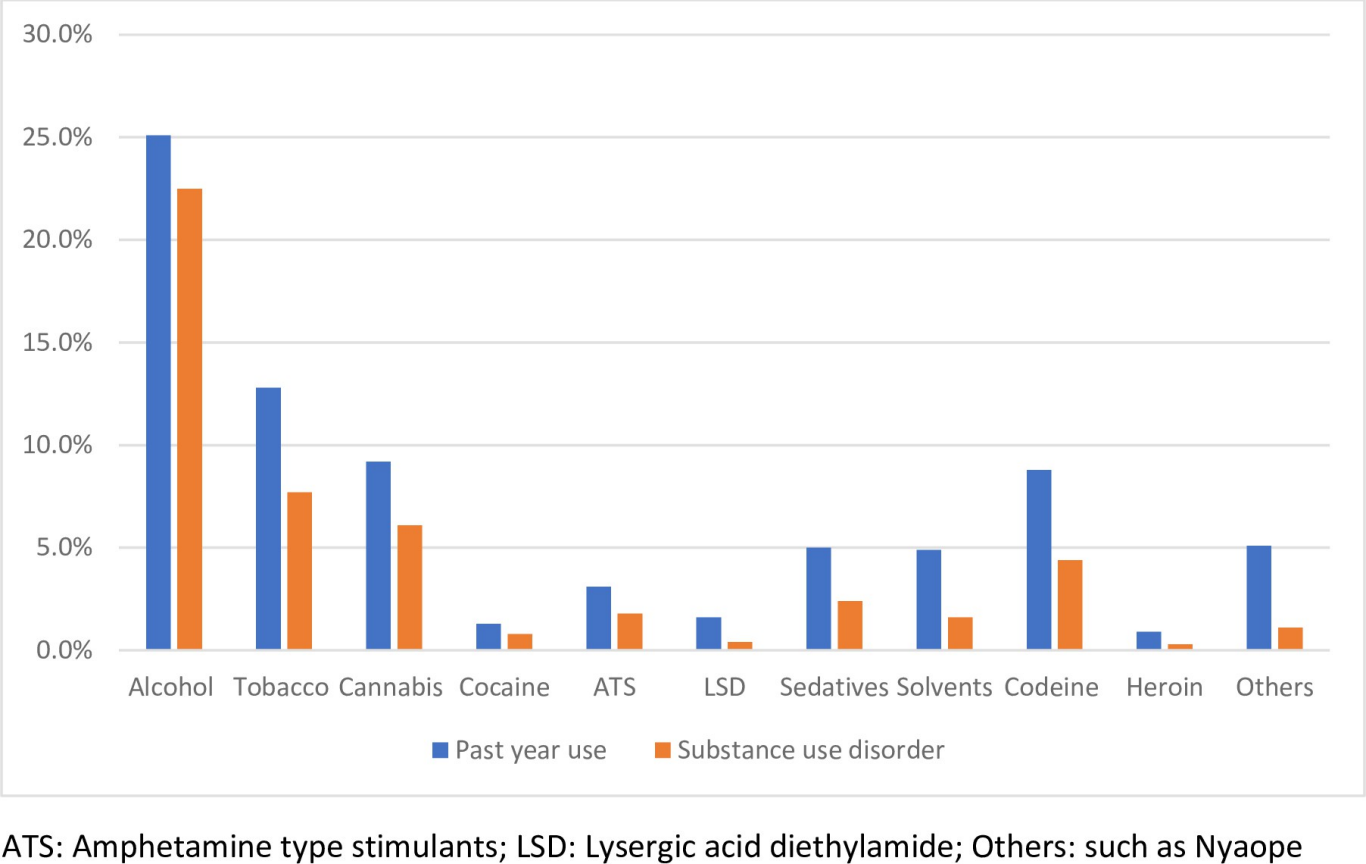
Pattern of psychoactive substance use among adolescent learners.

**Fig 2:**
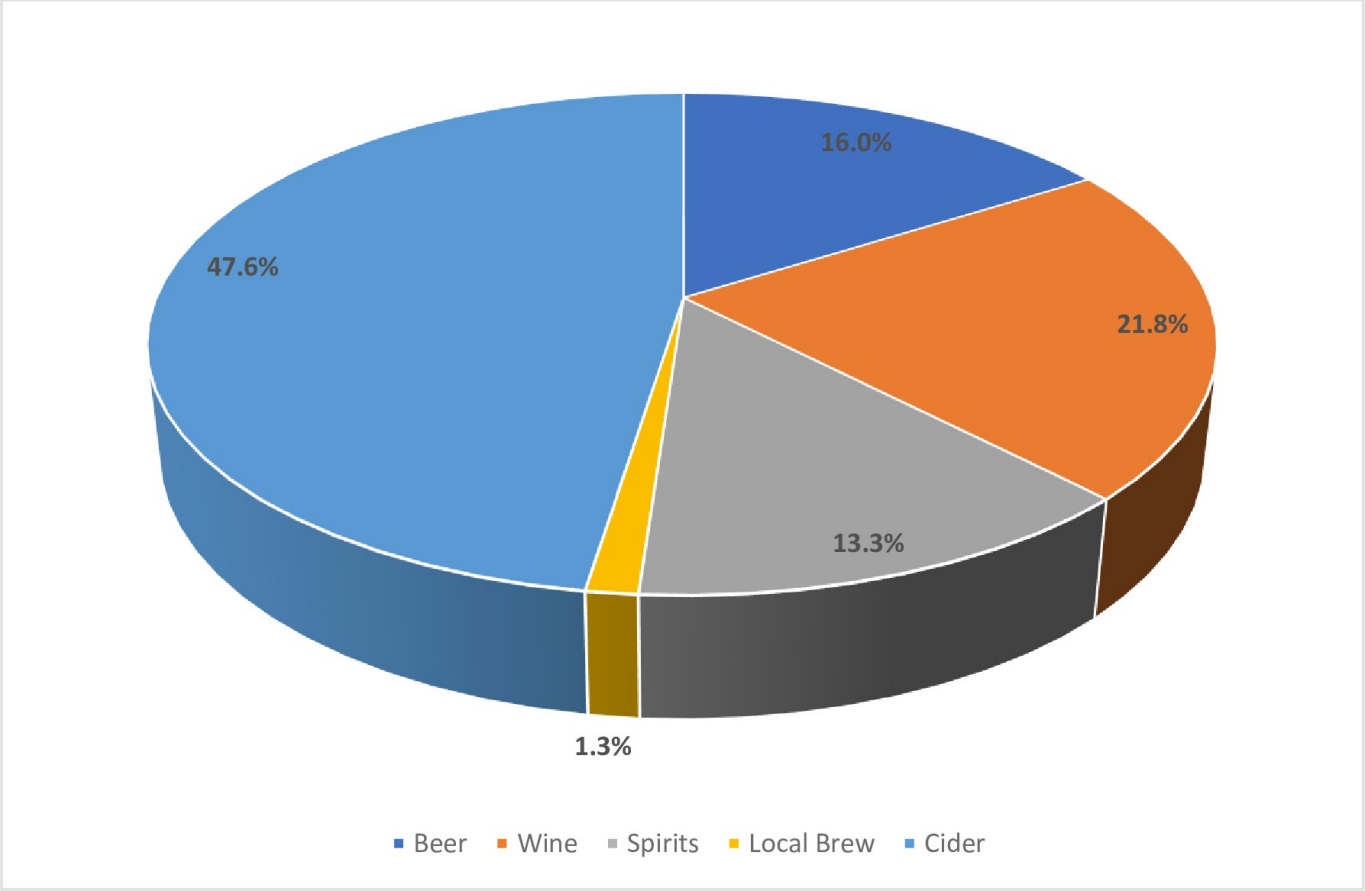
Types of alcohol products consumed by the adolescent learners.

Three hundred and thirty-one (44.6%) participants admitted using at least one psychoactive substance in the past 12 months, while 21% had used more than one substance in the same period. Alcohol was the most used psychoactive substance, with 25.1% of the previous year’s users (Fig 1). Cider (47.6%), wine (21.5%), and beer (16%) were the most frequently used alcohol products (Fig 2). Tobacco products (12.8%), in the form of cigarettes and snuff, were the second most used psychoactive substance, followed by cannabis (9.2%) (Fig 1). Inhalants such as petrol and glue were used by 4.9% of adolescents sampled, while codeine from cough syrup was used by 8.8% of participants (Fig 1). The amphetamine-type stimulant was commonly used in the form of CAT, M-CAT, or methcathinone, a monoamine alkaloid psychostimulant. Other drugs used included a mixture of psychoactive substances such as ‘Nyaope,’ a street drug commonly found in South Africa. The street cocktail drug, also known as ’Whoonga’ or ’Bluetooth,’ is a mixture of low-grade heroin, cannabis products, antiretroviral drugs, and other bulking agents. Two hundred and thirty-four (31.5%) of the participants met the criteria for SUDs according to DSM-5 criteria for at least one psychoactive substance.

### Factors associated with SUD

On bivariate analysis, factors that were significantly associated with SUD included older age (t = -4.40; p <0.01), male gender (χ2 = 9.97, p = 0.002), regular participation in activities (χ2 = 25.1, p <0.01) and having a friend (χ2 = 66.4, p <0.01), father (χ2 = 25.0, p <0.01) or mother (χ2 = 26.3, p <0.01) who uses psychoactive substance (Table 2). It was also observed that higher levels of PD or higher GHQ-12 score (t = -4.43; p <0.01) was significantly associated with SUD (Table 3).

**Table 2:** Associated factors of substance use disorder in the secondary school students.

Variables	Substance use disorder				Statistic		
	Absent		Present		*df*	*χ* ^2^	*p*
	Frequency	%	Frequency	%			
**Gender**							
Male	204	62.6	122	37.4	1	9.97	**0.002**
Female	299	73.5	108	26.5			
**Ethnic group**							
Non-citizen	1	100.0	-	-	3	6.95	0.73
Tswana	393	68.3	182	31.7			
Kalanga	78	78.0	22	22.0			
Other ethnic groups	21	56.8	16	43.2			
**Religion**							
No religious affiliation	37	63.8	21	36.2	3	0.85	0.839
Christianity	452	68.7	206	31.3			
Islam	3	75.0	1	25.0			
Others	11	73.3	4	26.7			
**The frequency of religious participation**							
Never or rarely	91	53.8	78	46.2	1	25.1	**<0.01**
Often or regularly	382	74.3	132	25.7			
**Loss of at least one parent to death or marital separation or divorce**							
No	10	55.6	8	44.4	1	1.41	0.236
Yes	492	68.7	224	31.3			
**Fathers’ occupation or employment status**							
Unemployed	81	64.8	44	35.2	1	1.69	0.431
Higher occupational level	65.8	84.3	81	34.2			
Lower occupational level	165	70.5	69	29.5			
**Mothers’ occupation or employment status**							
Unemployed	123	68.3	57	31.7	1	0.80	0.670
Higher occupational level	177	66.0	91	34.0			
Lower occupational level	126	70.0	54	30.0			
**Having a father who uses any psychoactive substance**							
No	313	75.8	100	24.2	1	25.0	**<0.01**
Yes	138	57.0	104	43.0			
**Having a mother who uses any psychoactive substance**							
No	404	73.6	145	26.4	1	26.3	**<0.01**
Yes	83	52.2	76	47.8			
**Having friends who uses any psychoactive substance**							
No	418	76.3	130	23.7	1	66.4	**<0.01**
Yes	72	49.2	226	31.6			

Significant p-values are in bold.

**Table 3:** The relationship between substance use disorder and the continuous variables.

Variables	SUD	Mean	SD	t	P
**Age**					
	No	15.10	1.610	-4.40	**<0.01**
	Yes	15.62	1.401		
**Monthly allowance**				-1.19	0.234
	No	18.96	18.250		
	Yes	20.98	21.590		
**GHQ score** *				-4.43	**<0.01**
	No	2.90	3.038		
	Yes	4.09	3.561		

Significant p-values are in bold

* Psychological distress score.

Table 4 shows the factors associated with SUD on the binary logistic regression model. Male gender (AOR [Adjusted odd ratio] = 1.94; 95% [confidence interval] CI: 1.26–2.995), having a friend (AOR = 4.27; 95% CI: 2.68–6.78), or father (AOR = 1.87; 95% CI: 1.14–3.04), who uses psychoactive substance, and higher levels of psychological distress (AOR = 1.09; 95% CI: 1.03–1.17) remained significantly associated with SUD in the secondary school students in Gaborone, Botswana. Regular participation in religious activities was protective (AOR = 0.61; 95% CI: 0.38 0.96) for SUD after a logistic regression analysis.

**Table 4:** Binary logistic regression model showing the predictors of SUD.

Characteristics	Wald	p-value	AOR	95% CI.	
				Lower	Upper
**Gender**					
Male	8.95	**0.003**	1.94	1.26	2.995
**Age**					
Older	1.67	0.197	1.11	0.95	1.29
**GHQ score**					
Higher	8.00	**0.005**	1.09	1.03	1.17
**Monthly upkeep in USD**					
Higher	<0.01	0.976	1.00	0.99	1.01
**Participation in religious activities**					
Regular	4.47	**0.034**	0.61	0.38	0.96
**Have you lost a parent to death or marital separation**					
Yes	1.14	0.286	2.51	0.46	13.6
**Do you have a friend using any substance?**					
Yes	37.6	**<0.01**	4.27	2.68	6.78
**Does your father use any substance?**					
Yes	6.24	**0.013**	1.87	1.14	3.04
**Does your mother use any substance?**					
Yes	1.32	0.250	1.39	0.79	2.41

Significant p values are in bold.

## Discussion

The main findings of this study were that a high proportion of learners (44.6%) used a psychoactive substance in the past 12 months, with at least 21% of learners using more than one psychoactive substance. Alcohol was the most used psychoactive substance, followed by tobacco and cannabis. This pattern is consistent with the findings of other studies in Africa [22–24], including Botswana [13] and international literature [2,25]. Parry and colleagues suggest that the choice of drug use in adolescent learners is influenced by factors such as age, peers, cost, and accessibility of illicit substances [22]. Unlike previously reported in Botswana in a survey that included all ages [16], the most common alcoholic beverage consumed by the adolescents in the present study was cider. This could be related to its taste and smell, as beer and spirit are bitter. Moreover, cider is mainly made from fruits such as apples, which makes it attractive, and the alcohol content is small, thus making it easy for youth to experiment with drinking.

Of the illicit psychoactive substances, cannabis was the most used amongst the participants of the current study, and it accounted for 9.2% of all the psychoactive substances, which is similar to the report from a previous study among the same population [14]. Of concern is the high prevalence of the non-medical use of codeine in cough syrup, which accounted for more than 8% of drug use among Botswana students. This trend has also been reported in other African countries [26,27], and the accessibility of this agent in the African communities, including Botswana, is fast becoming a source of concern. Codeine is usually mixed with soft drinks such as Sprite to form a concoction known on the street as “Purple drank or Lean” [28]. Adolescents use this method to mask the abuse of this substance so that they are not detected until they begin to show severe signs of SUD [28]. Since it may be very difficult to control the use of this substance, it is recommended that the importation of the cough mixture with codeine be banned, as was proposed in another African country with a similar problem [26]. Furthermore, the non-medical use of antiretroviral drugs, which adolescents mix with cannabis to form ‘Nyaope,’ is also concerning, as it may increase antiretroviral resistance apart from the potential danger of chemical interaction.

The high prevalence of adolescent substance use and the finding that almost one-third of participants met SUD criteria in this study are consistent with the literature [24,29]. This is a major public health concern, as it is associated with several adverse outcomes such as poor academic performance, including school dropout [23], increased risk of interpersonal violence, and motor vehicle accidents [30]. It is also associated with a greater probability of engaging in high-risk sexual behaviors, HIV infection [31], a higher risk for psychological distress, and mental health problems, including suicidal behaviour [13,22]. In addition, using more than one substance, as seen in 21% of participants, may further confound these complex and multi-directional relationships [13]. There is thus an urgent need to provide drug screening and age-appropriate substance use interventions integrated with school health services to comprehensively address the short- and long-term implications of substance use.

In the present study, SUD was associated with the male gender, having a friend or family member who uses substances, psychological distress, and increased frequency of religious participation after logistic regression. Gender issues in substance use have been widely reported, with males dominating the picture [2,11]; it is only reasonable that males would have more propensity to develop SUD than females, as revealed by our findings.

Peer influence is an essential associated factor of substance use in adolescents, and the current study again supported this, as having a friend that used psychoactive substances was associated with an increased likelihood of adolescent SUD [24,32]. This was further supported by a recent qualitative study in a South African township [23], which reported peer influence as a major contributor to adolescent substance use. Conversely, it should be noted that peer relationships may also positively influence substance use behavior if the peers are abstinent [33]. This finding suggests that substance prevention interventions should include socialization skills and peer pressure management to modify this factor.

The finding that parental or familial substance use was associated with SUD in this study was again consistent with the literature [13,34]. For example, Trucco [35], in a review of psychosocial factors associated with adolescent substance use, suggested that parents and peers have the most critical influence on adolescent substance use behavior. These findings may be due to the direct effect of access, like offering substances to adolescents, or indirect factors such as perception of familial substance use approval and modelling. This highlights the need to include family interventions when treating individuals with SUDs, particularly for substance users with teenage children, to prevent the cycle of substance use in successive generations. This is because family-focused therapy acknowledges the family’s role in the etiology and the maintenance of drug-related disorders, and it has the power to facilitate recovery [36]. In addition, it acknowledges the reciprocal influences that exist between patients and their families.

Previous studies have demonstrated an association between psychological distress and substance use [13,21,37], and this study similarly supported this. However, the nature of this association requires further investigation in longitudinal studies, as substance use may precipitate or exacerbate psychological distress, while psychological distress may drive adolescents to self-medicate with substance use [13]. In addition, the dual burden of substance use and mental illness is likewise associated with increased disability, morbidity, and mortality [38]. Hence, adolescents screening positive for substance use must also be screened for mental disorders, and those with mental illness must be screened and targeted in substance prevention programs.

Whilst the present study’s findings revealed no relationship between religious affiliation and SUD, a negative correlation was found between the frequency of participation in religious activities and SUD. Perhaps, this suggests that regardless of people’s religious affiliations, active participation in religious activities is protective against SUD. The negative correlation between active participation in religious activities and the likelihood of SUD has been extensively documented in Europe [39], America [40], and Africa, including Botswana [13]. Two hypotheses have been suggested to mediate this relationship: the social support hypothesis and the mental health mediation hypothesis [41]. The first type mediates the relationship by creating an atmosphere or social norms against drug use through stigma and association with non-drug-using buddies [41]. The second type suggests the use of strong religious beliefs as a coping mechanism against drug use. While the two theories seem plausible, they are yet to be substantiated [41]. Thus, more studies targeting the benefit of religious practice and how it can be crafted into substance prevention or intervention programs should be encouraged.

### Limitations

This study has some limitations in that it was a cross-sectional questionnaire survey, had a limited sample size, and lacked an objective measure of drug toxicology. The self-report nature of this study may have introduced some recall bias or over-reporting, but an effort was made to reduce these influences on the results. For example, a fictitious drug was added to the list of psychoactive substances, and those who claimed to have used this drug were removed from the analysis. In addition, the use of diagnostic interview tools to assess substance use disorder may have likewise strengthened the study. However, the study highlights the vital concern of adolescent psychoactive substance use and its associated factors in a developing setting.

## Conclusions

This study’s findings reinforce adolescents’ high vulnerability to use psychoactive substances in this critical developmental period and the need to acknowledge the vital role of peers, family, and parents in adolescents’ substance use behaviour. Health promotion and substance use interventions should include promoting positive role models and family-focused and religious-based interventions. The association between substance use and increased psychological distress also suggests the potential dual burden of disease among those with substance use disorder. This problem may be addressed by including integrated health services focusing on physical, mental, and SUD in the school system. Further longitudinal research among larger samples that include interventions for resource-limited settings is required.

## Abbreviations

SUDs: Substance Use Disorders
DSM-5: Diagnostic and Statistical Manual of Mental Disorders, fifth edition (DSM-5)
IRB: Independent Review Board
PD: Psychological Distress
